# 17‐β‐Estradiol Protects Chondrocytes From Senescence and Ameliorates Osteoarthritis Progression via ERα‐AKT‐FOXO4 Pathway

**DOI:** 10.1111/jcmm.71018

**Published:** 2026-02-12

**Authors:** Yikai Liu, Jiangshan Ai, Zian Zhang, Xinzhe Lu, Chaoqun Yu, Yejun Zha, Haining Zhang

**Affiliations:** ^1^ Department of Orthopaedics and Traumatology Beijing Jishuitan Hospital Affiliated to Capital Medical University Beijing China; ^2^ Department of Joint Surgery The Affiliated Hospital of Qingdao University Qingdao Shandong Province China; ^3^ Department of Thoracic Surgery, Shanghai General Hospital Shanghai Jiao Tong University School of Medicine Shanghai China; ^4^ Department of Orthopedic Trauma Zhengzhou Central Hospital Affiliated to Zhengzhou University Zhengzhou China

**Keywords:** 17‐β‐Estradiol, cellular senescence, chondrocyte, Forkhead box protein O4, osteoarthritis

## Abstract

Osteoarthritis (OA) is a prevalent cause of joint pain in elderly individuals, and chondrocyte senescence plays a crucial role in its pathogenesis. FOXO4 has been identified as a crucial molecule in cellular senescence. However, little is known regarding its role in OA and the regulation of its expression. 17‐β‐Estradiol (E2) has been demonstrated to exert a protective effect in OA, yet the underlying mechanism remains largely unexplained. In this study, we reported a protective effect of E2 against multiple types of chondrocyte senescence, and this effect was mediated by oestrogen receptor α (ERα). Mechanically, E2 activated AKT and facilitated the nuclear export and the degradation of FOXO4, which played a crucial role in resisting senescence. Moreover, knockdown of FOXO4 in osteoarthritic chondrocytes alleviated cellular senescence. Furthermore, we demonstrated that intra‐articular injection of E2 was effective in ameliorating surgery‐induced OA in a rat model. Collectively, E2 contributed to the alleviation of chondrocyte senescence through the ERα‐AKT‐FOXO4 signalling pathway and ameliorated OA progression in the rat model. Our study offers a novel therapeutic approach for controlling chondrocyte senescence and provides insights into the role of E2 in treating OA.

**Abbreviations**
DMM: destabilise the medial meniscus
E2: 17‐β‐Estradiol
FOXO4: Forkhead box protein O4
IAJ: intra‐articular injection
MOA: mild OA
OA: osteoarthritis
SASP: senescence‐associated secretory phenotype
SOA: severe OA

## 1 Introduction

Osteoarthritis (OA) is a common disease characterised by joint cartilage degeneration and pain, which seriously diminishes the quality of life of elderly individuals. The aging population increases the incidence of OA, and the cost of OA treatments accounts for 2% GDP in developed countries, imposing a substantial burden on elderly people [1]. Despite the increasing prevalence of the condition and the growing number of research studies on OA in recent years, therapeutic options remain limited, primarily focusing on symptom alleviation, such as painkillers and joint replacement [2]. Currently, there is an absence of disease‐modifying osteoarthritis (OA) drugs capable of ameliorating or delaying disease progression. Consequently, considerable efforts have been exerted in recent decades to identify novel potential therapeutic targets for OA [3]. Several existing conventional and biological disease‐modifying anti‐rheumatic drugs (DMARDs), such as hydroxychloroquine (HCQ) [4, 5], methotrexate (MTX) [6], tocilizumab [7], and interleukin (IL)‐1 inhibitors [8], which are typically utilised in the management of inflammatory rheumatic diseases, have been reconsidered for the treatment of OA. However, few of these drugs obtained satisfactory outcomes. Therefore, an effective way to prevent OA progression is urgently needed.

Chondrocytes are the only cell type in cartilage and directly affect the metabolism of the cartilage matrix [9]. Recently, chondrocyte senescence has been recognised as a crucial pathological process in OA development, and inhibiting chondrocyte senescence has begun to be considered as a therapeutic approach, even though the underlying mechanism remains largely unknown [10, 11, 12]. Cellular senescence is a state characterised by increased expression of p16 and p21, elevated levels of cellular SA‐β‐Gal, cell cycle arrest, resistance to apoptosis, and continuous secretion of senescence‐associated secretory phenotype (SASP) factors [13]. A relationship between senescence and OA has long been established [14, 15, 16], but it is only in recent years that senescence has started to be considered as a therapeutic target. An increasing number of newly identified drugs targeting senescent cells or SASP factors support the use of anti‐senescence therapy in OA [17, 18, 19, 20]. Selective clearance of senescent chondrocytes ameliorated OA progression in a surgery‐induced OA mouse model [21]. In contrast, injection of senescent cells into the joint cavity could induce an OA‐like state in mice [22]. These findings indicated that senescent cells contributed significantly to OA progression.

Forkhead box protein O4 (FOXO4) has been identified as a pivotal factor for the viability of senescent cells. FOXO4 binds to p53, upregulates p21 expression and ultimately leads to cell cycle arrest [23]. The synthetic peptide FOXO4‐DRI exhibits competitive inhibition against FOXO4, which can selectively eliminate senescent cells from in vitro‐expanded human chondrocytes by disrupting the p53‐FOXO4 interaction and triggering senescent cell apoptosis [24]. Furthermore, the FOXO4‐DRI can neutralise doxorubicin‐induced chemotoxicity and restore the functions of multiple organs in both prematurely aged mice and naturally aged mice [25]. Typically, FOXO4 is located in the nucleus; however, when phosphorylated, it is translocated to the cytoplasm and degraded in a ubiquitination‐dependent manner. The upstream PI3K‐AKT pathway can phosphorylate FOXO4, leading to its degradation [26].

17‐β‐Estradiol (E2) has been reported to exert a protective effect against chondrocyte apoptosis and cartilage injury [27, 28], which partly explains the increased incidence rate of OA after menopause. However, whether E2 can inhibit chondrocyte senescence and whether there exists a regulatory relationship between E2 and FOXO4 in OA remain unclear to date. In our study, we found that E2 alleviated various forms of cellular senescence and exerted protective effects via the ERα‐AKT‐FOXO4 axis in chondrocytes. The incidence of OA in postmenopausal women has risen markedly, and this increase may be linked to the withdrawal of oestrogen. Unfortunately, currently, there are no drugs specifically targeting this mechanism for the treatment of OA, nor is there a sufficient understanding of the underlying mechanisms involved. Consequently, gaining a deeper insight into the mechanisms of E2 on chondrocytes and cartilage is of paramount importance. Our study provides a therapeutic approach for controlling chondrocyte senescence and inhibiting OA progression through E2 administration and demonstrates the harmful effects of FOXO4 in chondrocyte senescence. Our findings indicate that oestrogen replacement therapy (ERT) holds potential in mitigating the progression of OA.

## 2 Materials and Methods

### 2.1 Human Samples

**FIGURE 1:**
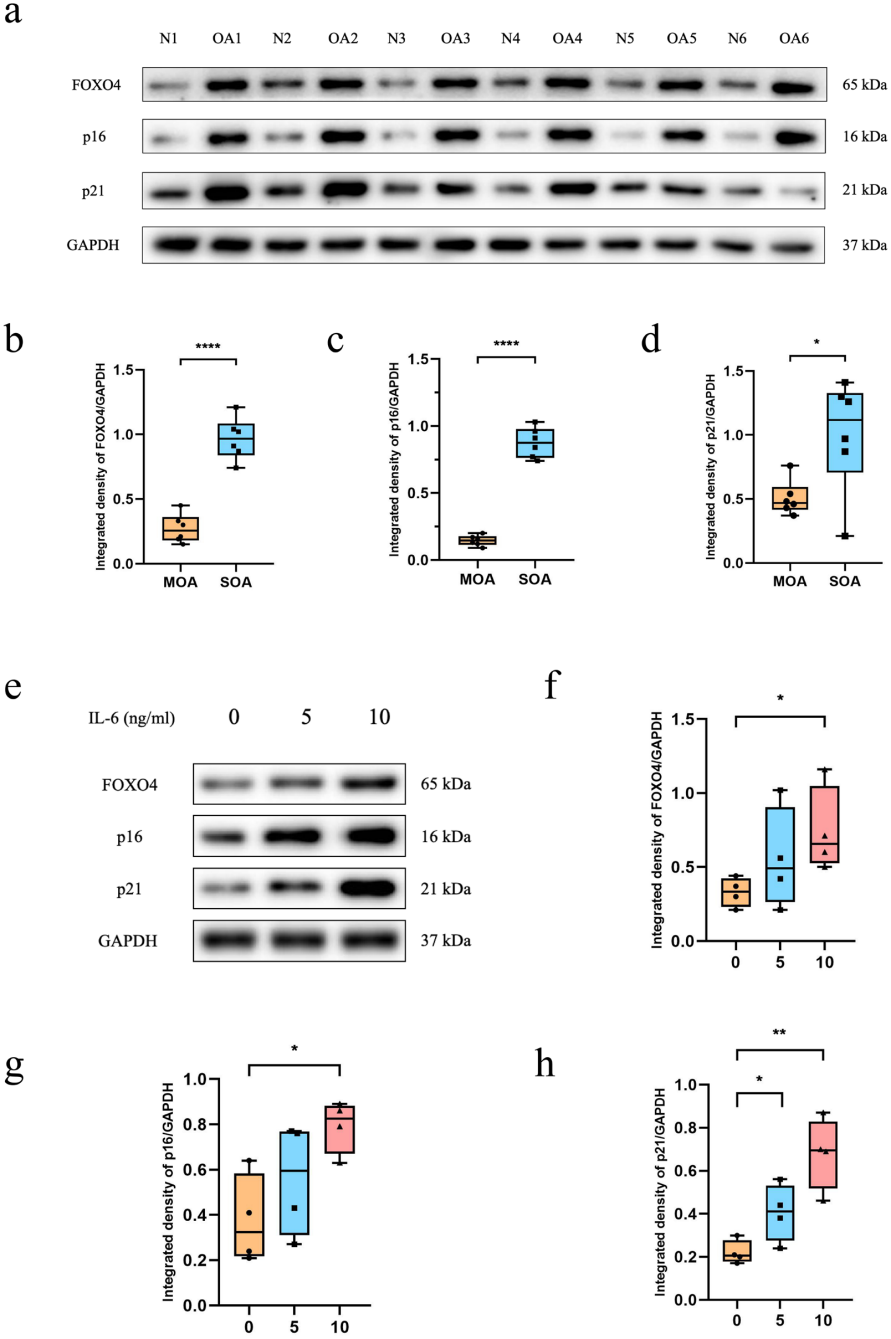
Osteoarthritic cartilage expresses higher levels of FOXO4 and senescence marker p16 and p21 (a‐d), and IL‐6 treatment of normal chondrocytes increased the expression of FOXO4 and p16 and p21 (e–h). (a) Normal and osteoarthritic cartilage was obtained, and Western blotting analysis of FOXO4, p16 and p21 expression was performed (cartilage was collected from 6 donors without OA and 6 donors with OA). (b–d) The relative levels of FOXO4 (b), p16 (c), and p21 (d) of cartilage collected from different donors are shown in the graphs. (e) Western blotting analysis of p16, p21, and FOXO4 expression in healthy chondrocytes after treatment with different concentrations of IL‐6 (*n* = 4) was conducted. (f–h) The relative levels of FOXO4 (f), p16 (g), and p21 (h) following treatment with varying concentrations of IL‐6 are presented. MOA: Mild OA; SOA: Severe OA. Student's *t* test was used to compare differences between two groups. For comparisons among multiple groups, one‐way analysis of variance (ANOVA) was applied, followed by Bonferroni's post hoc test for multiple comparisons. A *p*‐value of less than 0.05 was considered statistically significant. All data are presented as the mean ± SD. *p < 0.05, **p < 0.01, ****p < 0.0001.

The study was approved by the ethics committee of the Affiliated Hospital of Qingdao University (QYFY WZLL 27764), and informed consents were obtained from all donors. Osteoarthritic cartilage samples were harvested from 12 female patients with OA undergoing total knee arthroplasty, aged 58–77 years (mean ± SD: 66.75 ± 6.84). Normal cartilage samples were obtained from 20 females undergoing amputation, with no history of OA or other knee diseases, aged 34–60 years (mean ± SD: 47.70 ± 8.10). To minimise age‐related bias, a 1:1 case–control matching was conducted using the SPSS software, with calliper values standardised to ensure a maximum age difference of 5 years between matched pairs. Ultimately, 6 pairs of patients were successfully matched, and their cartilage samples were used to compare the expression of p16, p21 and FOXO4, as shown in Result 1 and Figure 1. The remaining cartilage samples were used for isolating and culturing osteoarthritic chondrocytes and healthy chondrocytes. Cartilage was collected from the medial femoral condyles, with OA specimens dissected from the area surrounding the cartilage lesions (within 2 cm).

### 2.2 Cell Culture

A portion of the cartilage was used to isolate chondrocytes. The cartilage was first washed with PBS, then minced into 1mm^3^ pieces, and subsequently digested for 6 h with collagenase II (Sigma, USA) on an orbital shaker at 37°C. The digested material was filtered through a 70‐μm cell strainer (Biologix, USA). After washing with high glucose DMEM (BI; Israel) supplemented with 1% penicillin/streptomycin (HyClone, USA), the freshly isolated chondrocytes were resuspended in high glucose DMEM containing 10% fetal bovine serum (Gibco, USA) and 1% penicillin/streptomycin. The cells were seeded at a density of 4 × 10^4^ cells/cm^2^, and incubated with 5% CO_2_ at 37°C. The media were replaced 24 h after seeding to remove the non‐adherent cells and were then replaced every 3 days. When the monolayer culture reached 80%–90% confluence, the cells were detached by trypsin and reseeded at 2 × 10^4^ cells/cm^2^ (P1) in a new flask or 6‐well plate. Only low‐passage cells (P2‐P3) were used in subsequent experiments.

### 2.3 In Vitro Reagent Treatment and RNA Interference

When the normal chondrocytes reached 70% confluence, the culture medium was changed and replaced with medium supplemented with different concentrations of IL‐6 (MCE, China, HY‐P7044G) to stimulate the chondrocytes. A treatment with 10 ng/mL IL‐6 for 24 h was selected for the induction of chondrocyte senescence [29, 30]. To alleviate senescence and observe the localisation of FOXO4, 10 nM E2 (Sigma‐Aldrich, USA, E8875) was used for 24 h [31]. To block ERα, 20 μM TPBM (MCE, HY‐131404) was used for 24 h [32]. To block ERβ, 1 μM PHTPP (MCE, HY‐103456) was used for 24 h. To block GPER, 1 μM G15 (MCE, HY‐103449) was used for 48 h [32, 33, 34, 35]. To inhibit AKT activity, 5 μM perifosine (MCE, HY‐50909) was used for 6 h [36]. Cellular senescence was induced using 20 μM etoposide for 48 h (Sigma‐Aldrich, E1383) [37], 200 μM H_2_O_2_ for 7 days [38], and 5 ng/mL IL‐1β for 24 h [39]. After treatment with the reagents, the cells were further incubated in fresh culture medium for 48 h before the final analysis.

For siRNA transfection, siRNA targeting either FOXO4 or ERα was obtained from RiboBio (China). The transfection was performed using a riboFECT CP Transfection Kit (RiboBio), following the manufacturer's instructions. The efficiency of transfection was confirmed by quantitative RT‐qPCR 48 h after transfection. The selected target sequence for the FOXO4 gene was 5′‐ATCTAGGTCTATGATCGCG‐3′, and the selected target sequence for the ERα gene was 5′‐GAGACTTGAATTAATAAGTGA‐3′. Additionally, a universal sequence, 5′‐TTCTCCGAACGTGTCACGT‐3′ was used as a negative control for RNA interference.

### 2.4 Western Blotting

A total of 100 μL RIPA buffer, supplemented with 2% PMSF (Thermofisher, USA), was added to the cells in each well of 6‐well plates. The chondrocytes were lysed on ice for 40 min to extract total protein. The total protein concentration and loading quantity were determined by BCA assays. Nuclear and cytoplasmic proteins were extracted using a commercially available kit (Beyotime, P0028, China). The proteins were then separated by sodium dodecyl sulfate–polyacrylamide gel electrophoresis (SDS‐PAGE) and transferred to polyvinylidene difluoride (PVDF) membranes under constant current conditions. Subsequently, the membranes were blocked with 5% skim milk at room temperature for 1.5 h. The membranes with proteins were incubated with primary antibodies overnight at 4°C. The primary antibodies used were rabbit anti‐human p21 (ab109520), p16 (ab270058), FOXO4 (ab128908, ab208620), ER (ab92516), and p‐FOXO4 (ab126594), TBP (ab818), as well as mouse anti‐human GAPDH (ab8245) and β‐tubulin (ab7291) primary antibodies (dilution concentration 1:1000 or 1:2000 according to the manufacturer's instructions), all purchased from Abcam, UK. Rabbit anti‐human p‐AKT (4060S) primary antibodies were purchased from Cell Signalling Technology, USA. Goat anti‐rabbit or goat anti‐mouse horseradish peroxidase‐conjugated secondary antibodies (dilution concentration 1:10000, Cell Signalling Technology, Boston, USA) were then added and incubated at room temperature for 1 h. Target proteins were detected by enhanced chemiluminescence reagent. The densitometry of the bands was analysed using ImageJ software.

### 2.5 Senescence‐Associated β‐Galactosidase Staining

SA‐β‐Gal activity was measured using a staining kit from Cell Signalling Technology (USA), following the manufacturer's protocols. The SA‐β‐Gal staining was performed in 6‐well plates. After discarding the culture medium and washing the cells with PBS three times, the chondrocytes were fixed for 15 min at room temperature, washed, and then incubated with the staining solution overnight at 37°C. The percentage of SA‐β‐Gal‐positive cells in three random fields was quantified and averaged using an Olympus microscope (Japan) at a magnification of 100× [40, 41].

### 2.6 Immunofluorescence (IF) Staining

Chondrocytes were seeded on cell climbing sheets in 24‐well plates for three days prior to IL‐6 or E2 treatment. Then, the climbing sheets were removed and the cells were fixed with 4% paraformaldehyde containing 0.03% Triton X‐100 for 2 h. After washing with PBS three times, the cells were blocked with bovine serum albumin for 30 min, followed by incubation with rabbit anti‐human FOXO4 antibodies (Abcam, UK) overnight at 4°C. Subsequently, the cells were incubated with Alexa Fluor 546‐conjugated goat anti‐rabbit antibodies (Invitrogen, USA) at room temperature for 1 h. The nuclei were stained with DAPI (Invitrogen, USA) for 5 min, and images were captured using a microscope (Olympus, Japan).

### 2.7 Flow Cytometry

Cell cycle progression was analysed by flow cytometry. After IL‐6 or E2 treatment, the cells were washed three times with 1 mL of PBS and then fixed with precooled anhydrous ethanol at 4°C overnight, following the instructions of the Cell Cycle and Apoptosis Kit (Beyotime Biotechnology, China). Each sample was resuspended in 500 μL of staining buffer supplemented with 10 μL of RNase (Beyotime, China) and 25 μL of PI staining solution (Beyotime, China). The cells were incubated at 37°C for 30 min. Red fluorescence was detected by CytoFLEX (Beckman, USA) at an excitation wavelength of 488 nm, using the PE‐H channel. The data analysis was conducted using FlowJo v10 software.

### 2.8 Enzyme‐Linked Immunosorbent Assay (ELISA)

MMP‐13 levels in the supernatants were determined with a commercially available ELISA kit from Wuhan Boster Biotechnology (China) according to the manufacturer's instructions. Results are expressed as mean ± standard deviation and represent data from three separate experiments.

### 2.9 Rats

Eight‐week‐old male Wistar rats were purchased from the Charles River Experimental Animal Center (Beijing, China). At 12 weeks of age, surgery to destabilise the medial meniscus (DMM) was performed to establish a rat model of OA, as previously described [42]. Prior to surgery, the rats were anaesthetised via intraperitoneal injection of pentobarbital sodium (40 mg/kg, UK). Sham surgery involved opening and exposing the joint cavity of the knee, followed by suturing the incision without causing any injury to the anterior cruciate ligament or meniscus in age‐matched rats. In the E2 intra‐articular injection (IAJ) group, E2 (5 μg/kg) was administered by intra‐articular injection once per week starting after surgery. In the sham group and control group, normal saline was administered instead. The rats were sacrificed at 20 weeks of age.

### 2.10 Histologic and Immunohistochemical Analyses

Rat cartilage was harvested at 8 weeks after surgery. After cardiac perfusion with 100 mL of normal saline and 10 mL of 4% paraformaldehyde (PFA), the knees were resected and incubated in 4% PFA for one week to fix the tissues. This was followed by decalcification using EDTA at pH 7.4 for 4 weeks at 37°C. After dehydration, all tissues were embedded in paraffin and sectioned at a thickness of 5 μm per layer in the sagittal plane. The tissues were dewaxed and hydrated prior to staining with Safranin O. Subsequently, paraffin embedding, sectioning and Safranin O staining were performed by Servicebio (China). Articular cartilage degeneration was graded using the Osteoarthritis Research Society International (OARSI)‐modified Mankin criteria [43, 44]. Cartilage thickness was measured in each 1000× visual field.

### 2.11 Statistical Analysis

SPSS 26.0 (IBM, USA) was used for statistical analysis. Data are expressed as the mean ± standard deviation (SD). Student's *t*‐test was used to compare differences between two groups. For comparisons among more than two groups, one‐way analysis of variance (ANOVA) was employed, followed by Bonferroni's post hoc test for multiple comparisons. A *p*‐value of less than 0.05 was considered statistically significant.

## 3 Results

### 3.1 Higher Levels of p16, p21, and FOXO4 Were Observed in Osteoarthritic Cartilage Compared to Normal Cartilage

We measured the gene expression of p16 and p21 in normal and osteoarthritic cartilage to investigate the relationship between senescence and the severity of OA. The expression levels of p16 and p21 in the OA group were markedly increased compared with those in the normal group (Figure 1a,c,d). To investigate whether the expression of FOXO4 was altered in OA, we also measured the expression of FOXO4 in these two groups. The expression level of FOXO4 in the OA group was markedly increased compared with that in the normal group (Figure 1a,b), suggesting a potential correlation among FOXO4, cellular senescence, and OA.

### 3.2 IL‐6 Induced Chondrocyte Senescence and FOXO4 Upregulation in a Dose‐Dependent Manner

We then aimed to investigate whether chondrocytes isolated and cultured in vitro could also express high levels of FOXO4 under pro‐senescence conditions. As IL‐6 is a well‐established SASP factor known to induce cellular senescence [29, 30], we first investigated the efficacy of IL‐6 in inducing chondrocyte senescence. Chondrocytes were isolated from the knee cartilage of patients who underwent amputation to obtain healthy chondrocytes. These cells were subsequently treated with 0, 5, or 10 ng/mL IL‐6. We found that the expression of p16 and p21 was upregulated with increasing concentrations of IL‐6, indicating that IL‐6 induced chondrocyte senescence in a dose‐dependent manner. Specifically, the expression of p16 in the 10 ng/mL group and the expression of p21 in both the 5 ng/mL group and 10 ng/mL group were increased compared with those in the 0 ng/mL group (Figure 1e,g,h). Since 10 ng/mL IL‐6 induced more pronounced cellular senescence, as evidenced by the elevated levels of p16 and p21, this concentration was selected for inducing chondrocyte senescence in subsequent experiments.

We then measured the expression of FOXO4 after treatment with different concentrations of IL‐6. Notably, the expression of FOXO4 in the 10 ng/mL IL‐6 group was significantly upregulated compared with that in the 0 ng/mL group (Figure 1e,f), suggesting a potential link between chondrocyte senescence and FOXO4.

### 3.3 Knockdown of FOXO4 Alleviated Osteoarthritic Chondrocyte Senescence

**FIGURE 2:**
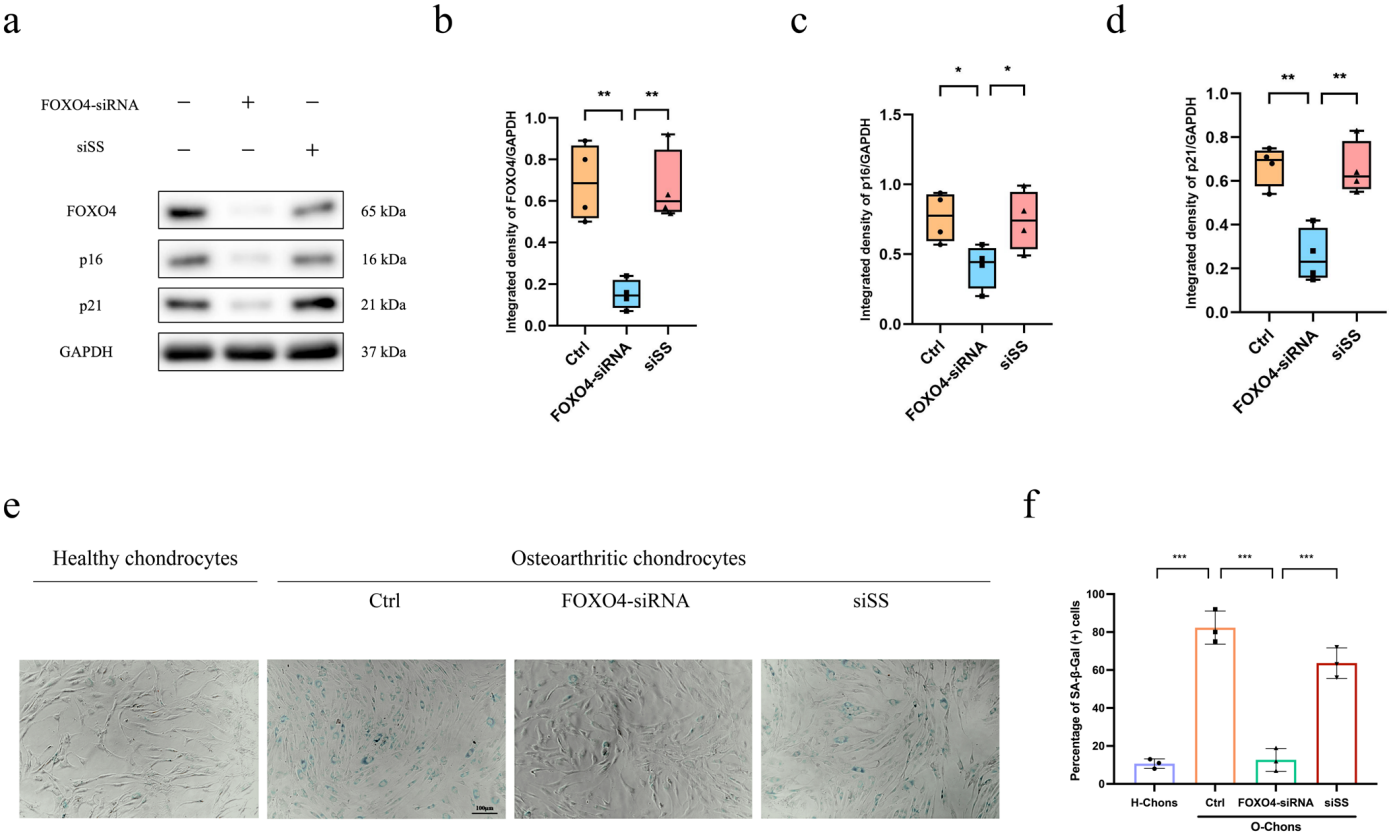
Knockdown of FOXO4 alleviated senescence in osteoarthritic chondrocytes. (a) Western blotting was performed to assess the expression of FOXO4, p16, and p21 in osteoarthritic chondrocytes, both with and without transfection of either FOXO4‐specific siRNA or a scrambled sequence siRNA (*n* = 4). (b–d) The relative levels of FOXO4 (b), p16 (c), and p21 (d) in osteoarthritic chondrocytes following transfection with either FOXO4‐specific siRNA or a scrambled sequence siRNA are shown. (e) Representative micrographs of SA‐β‐Gal staining are presented for healthy chondrocytes, as well as osteoarthritic chondrocytes transfected with FOXO4‐specific siRNA or a scrambled sequence siRNA (*n* = 3). (f) Quantification of SA‐β‐Gal (+) cells in these four groups. siSS: Scrambled sequence of siRNA as a control. H‐Chons: Healthy chondrocytes; O‐Chons: Osteoarthritic chondrocytes. One‐way analysis of variance (ANOVA) was applied, followed by Bonferroni's post hoc test for multiple comparisons. A *p*‐value of less than 0.05 was considered statistically significant. All data are presented as the mean ± SD. *p < 0.05, **p < 0.01, ***p < 0.001.

To explore whether FOXO4 plays a pivotal role in chondrocyte senescence, FOXO4‐specific siRNA was transfected into osteoarthritic chondrocytes to knockdown FOXO4 expression. The levels of FOXO4 protein were markedly decreased after FOXO4‐siRNA transfection compared with scrambled RNA transfection (Figure 2a,b), suggesting successful knockdown of FOXO4. Additionally, the protein levels of p16 and p21 in the FOXO4‐siRNA group were also reduced (Figure 2a,c,d). Furthermore, according to the SA‐β‐Gal assays, the percentage of positively stained cells in the FOXO4‐siRNA group was significantly decreased compared with those in the other two groups (Figure 2e,f). These findings indicate that FOXO4 is crucial in chondrocyte senescence in OA.

### 3.4 E2 Could Alleviate IL‐6‐Induced Chondrocyte Senescence

**FIGURE 3:**
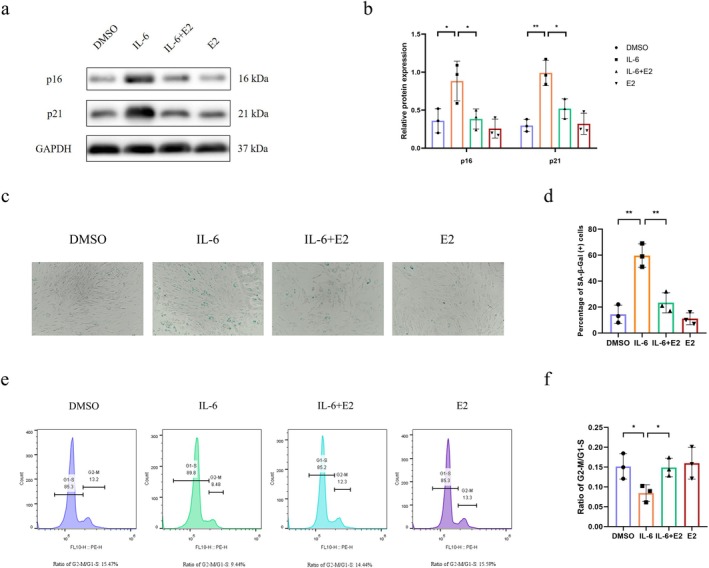
E2 could alleviate IL‐6‐induced chondrocyte senescence. Normal chondrocytes were used in this section. (a) Western blotting was performed to assess the expression of p16 and p21 after treatment with IL‐6 and/or E2 (*n* = 3). (b) The relative levels of p16 and p21 in these four groups are shown in the graph. (c) Representative micrographs of SA‐β‐Gal staining following treatment with IL‐6 and/or E2 (*n* = 3). (d) Quantification of SA‐β‐Gal (+) cells in the four groups is provided. (e) Cell cycle distribution was measured by flow cytometry after treatment with IL‐6 and/or E2 (*n* = 3). (f) The ratio of cells in the G2‐M to G1‐S phases in the four groups is shown. One‐way analysis of variance (ANOVA) was applied, followed by Bonferroni's post hoc test for multiple comparisons. A *p*‐value of less than 0.05 was considered statistically significant. All data are presented as the mean ± SD. *p < 0.05, **p < 0.01.

Postmenopausal women have a higher incidence of OA, suggesting a protective effect of E2 against OA progression. Therefore, we next investigated whether E2 can alleviate the senescence induced by IL‐6 and explored the role of FOXO4 in this process. Chondrocytes used in this section were isolated from normal cartilage. Four groups were established: the control group, the IL‐6 group, the IL‐6 + E2 group, and the E2 group. The expression of p16 and p21 in the IL‐6 group was higher than that in the control group, indicating the effectiveness of IL‐6 in inducing chondrocyte senescence. The upregulation of p16 and p21 expression was reversed by E2 (Figure 3a,b). The percentage of SA‐β‐Gal positive cells in the IL‐6 group was markedly increased compared with that in the control group, and E2 treatment successfully decreased the percentage of SA‐β‐Gal (+) cells (Figure 3c,d). Additionally, one feature of cellular senescence is cell cycle arrest, characterised by a low ratio of cells in the G2/S phase compared to those in the G1/M phase. We then used flow cytometry to analyse the distribution of cells throughout the cell cycle in the different groups. The G2‐S/G1‐M cell ratio decreased after IL‐6 treatment, whereas E2 treatment reversed this decrease (Figure 3e,f). Furthermore, we assessed the expression of γ‐H2AX, a well‐established biomarker for DNA damage and cellular senescence. Compared to the DMSO control, IL‐6 treatment significantly enhanced γ‐H2AX fluorescence intensity. This IL‐6‐induced increase was effectively attenuated by E2 treatment (Figure S1a,b). To further confirm the anti‐senescence effects of E2, we evaluated the levels of MMP‐13, a key SASP factor associated with cartilage matrix degradation, in the culture supernatant. As expected, IL‐6 markedly upregulated MMP‐13 levels, an effect that was rescued by E2 treatment (Figure S1c). These findings suggest an antisenescence effect of E2 in IL‐6‐induced chondrocyte senescence.

### 3.5 E2 Promoted the Phosphorylation and the Nuclear Export of FOXO4 by Activating AKT

**FIGURE 4:**
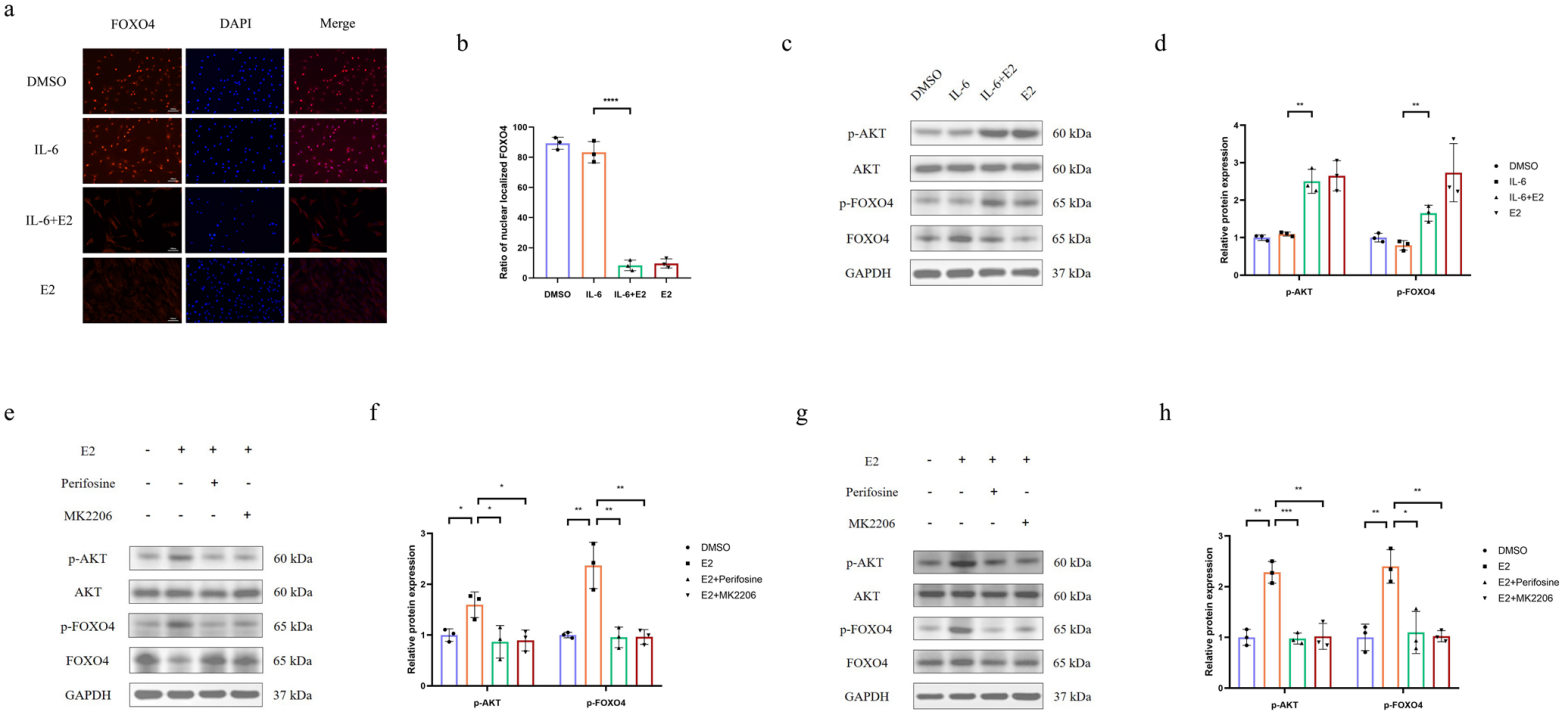
E2 promoted the phosphorylation and the nuclear export of FOXO4 by activating AKT. Normal chondrocytes were used for (a–d, g, h) and osteoarthritic chondrocytes were used for (e, f). (a) Immunofluorescence staining of FOXO4 was performed after treatment with IL‐6 and/or E2 (*n* = 3). (b) The ratio of nuclear‐localised FOXO4 is shown. (c) Western blotting was conducted to assess the phosphorylation levels of AKT and FOXO4 after treatment with IL‐6 and/or E2 (*n* = 3). (d) The relative levels of p‐AKT and p‐FOXO4 are presented in the graph. (e, g) Western blotting was performed to assess the phosphorylation levels of AKT and FOXO4 in response to AKT inhibition. Osteoarthritic chondrocytes (e) and healthy chondrocytes (g) were treated with the inhibitors perifosine or MK2206, as indicated (*n* = 3). (f, h) The relative levels of p‐AKT and p‐FOXO4 in the graph are provided. One‐way analysis of variance (ANOVA) was applied, followed by Bonferroni's post hoc test for multiple comparisons. A *p*‐value of less than 0.05 was considered statistically significant. All data are presented as the mean ± SD. *p < 0.05, **p < 0.01, ***p < 0.001.

We noticed that the subcellular localisation of FOXO4 changed in the immunofluorescence assay. IL‐6 treatment did not alter FOXO4 localisation, whereas E2 treatment induced its nuclear export (Figure 4a,b). Moreover, Western blot analysis of FOXO4 levels in the nuclear and cytoplasmic fractions showed that its nuclear expression was markedly reduced after E2 treatment, while its cytoplasmic expression increased (Figure S2). Typically, FOXO4 is a transcription factor located in the nucleus, and its phosphorylation and subsequent nuclear export indicate degradation. AKT is a kinase that acts upstream of FOXO4 and plays an indispensable role in the phosphorylation of FOXO4 [45, 46].

We then measured the levels of phosphorylated FOXO4 and AKT. The levels of phosphorylated FOXO4 and AKT did not change in response to IL‐6 treatment, but increased in the presence of E2 (Figure 4c,d). These findings indicated that FOXO4 was phosphorylated and subsequently exported from the nucleus under E2 treatment conditions, and this was related to the phosphorylation of AKT upstream.

We found that E2 promoted AKT and FOXO4 phosphorylation, but whether FOXO4 was phosphorylated by phosphorylated AKT remained unclear. Osteoarthritic chondrocytes (Figure 4e,f) and healthy chondrocytes (Figure 4g,h) were used to explore the mechanism of FOXO4 phosphorylation. We used Perifosine or MK2206, two AKT inhibitors, in combination with E2 to eliminate the effect of AKT phosphorylation. As expected, the levels of phosphorylated AKT in the E2 + perifosine group and E2 + MK2206 group were significantly lower than those in the E2 group, suggesting the effectiveness of perifosine and MK2206 in preventing AKT phosphorylation. Similarly, the levels of phosphorylated FOXO4 in the E2 + perifosine group and the E2 + MK2206 group were markedly reduced compared with those in the E2 group, indicating that AKT phosphorylation was indispensable for FOXO4 phosphorylation under E2 treatment conditions (Figure 4e–h).

To corroborate these findings, we assessed FOXO4 degradation dynamics using a cycloheximide chase assay with or without proteasome inhibition. E2 treatment promoted rapid FOXO4 decay within 2–4 h. This reduction was reversed by the proteasome inhibitor MG‐132, indicating that E2 promotes FOXO4 degradation via the proteasomal pathway (Figure S3).

### 3.6 E2 Exerted an Antisenescence Effect via ERα, Not ERβ or GPER

**FIGURE 5:**
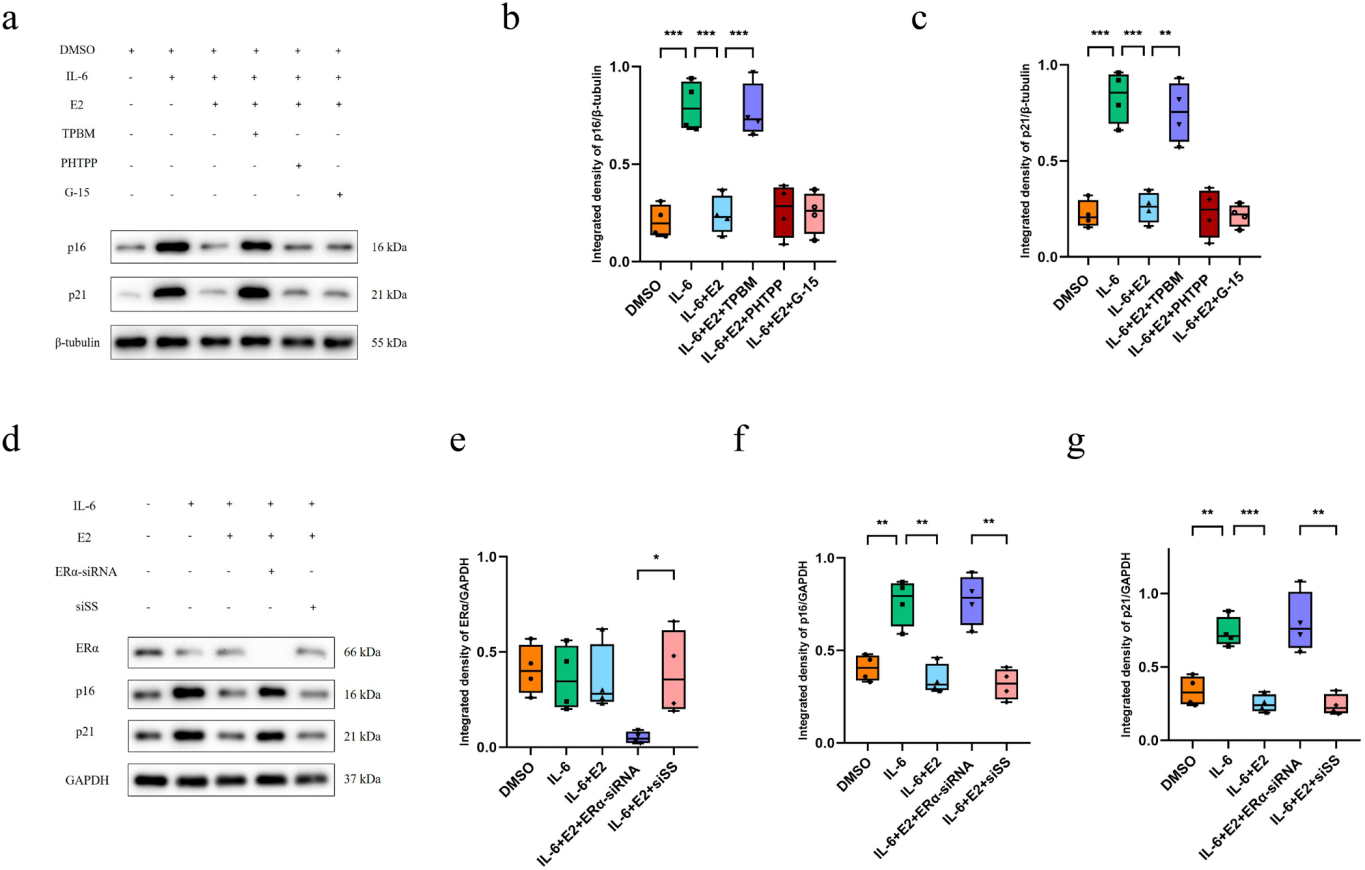
E2 exerted an antisenescence effect via ERα, not ERβ or GPER. Normal chondrocytes were used in this section. (a) Western blotting was performed to assess the expression of p16 and p21 after treatment with several combinations of IL‐6, E2, TPBM, PHTPP and G‐15 (*n* = 4). (b) The relative levels of p16 and p21 in the six groups are shown. (c) Western blotting was conducted to assess the expression of ERα, p16 and p21 after treatment with IL‐6 and/or E2 with or without ERα‐specific siRNA transfection (*n* = 4). (d–g) The relative levels of ERα (e), p16 (f) and p21 (g) in the five groups are presented. One‐way analysis of variance (ANOVA) was applied, followed by Bonferroni's post hoc test for multiple comparisons. A *p*‐value of less than 0.05 was considered statistically significant. All the data are presented as the mean ± SD. *p < 0.05, **p < 0.01, ***p < 0.001.

There are three common receptors of E2: ERα, ERβ and GPER [47, 48]. However, the role of each receptor in the antisenescence effect of E2 remains unknown. To identify the role of these receptors in resisting cellular senescence, we conducted the following experiments. Normal chondrocytes were collected and IL‐6 was used to induce cellular senescence. First, we used TPBM, a small molecule that blocks ERα; PHTPP to block ERβ; and G‐15 to block GPER. The expression of p16 and p21 was markedly increased after IL‐6 treatment compared with the DMSO group and could be reversed by E2 treatment (Figure 5a–c). Notably, when ERα was blocked with TPBM, the expression of p16 and p21 returned to high levels again, showing a significant difference from those in the IL‐6 + E2 group (Figure 5a–c). However, blocking ERβ or GPER caused no significant changes in the expression of p16 and p21 (Figure 5a–c). These findings indicated that ERα, but not ERβ or GPER, mediated the antisenescence effect of E2. To further confirm this, we transfected ERα‐specific siRNA into normal chondrocytes and measured the expression of ERα, p16 and p21. The expression of ERα was markedly decreased in the ERα‐siRNA group compared with the scrambled sequence transfection group, suggesting effective knockdown of ERα (Figure 5d,e). The expression of p16 and p21 was elevated under IL‐6 treatment conditions and returned to normal after treatment with both IL‐6 and E2 (Figure 5f,g). As expected, when transfected with ERα‐siRNA, the expression of p16 and p21 was significantly increased compared with that in the scrambled sequence transfection group (Figure 5f,g), indicating that the protective effect of E2 against senescence was completely abrogated.

### 3.7 E2 Could Alleviate Chondrocyte Senescence Induced by IL‐1β or H_2_O_2_ , but Could Not Alleviate Chondrocyte Senescence Induced by Ion Radiation or Etoposide Treatment

**FIGURE 6:**
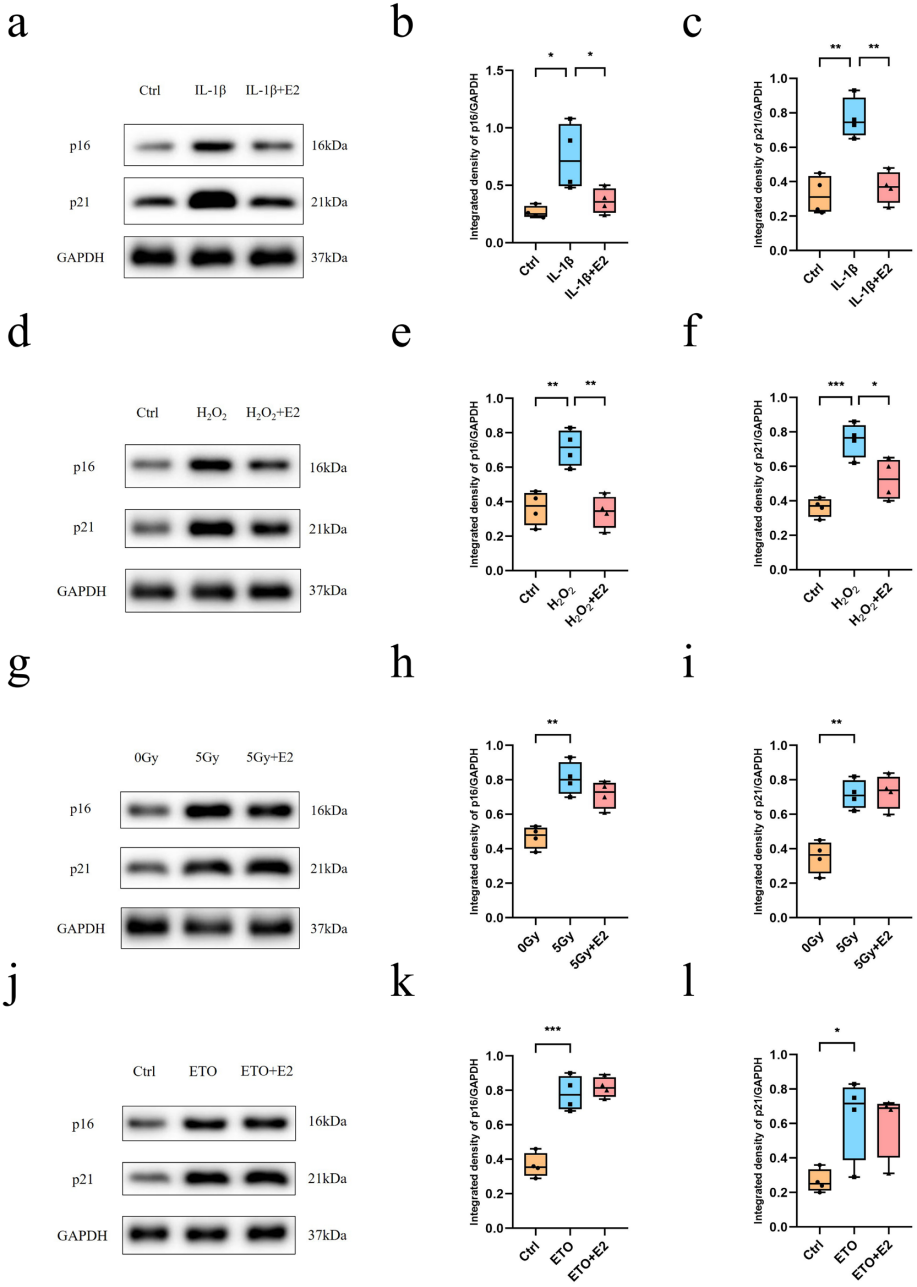
E2 could alleviate chondrocyte senescence induced by IL‐1β or H_2_O_2_, but it could not alleviate chondrocyte senescence induced by ion radiation or etoposide treatment. Normal chondrocytes were used in this section. (a) Western blotting was performed to assess the expression of p16 and p21 in chondrocytes treated with IL‐1β, with or without E2 (*n* = 4). (b, c) The expression levels of p16 (b) and p21 (c) in chondrocytes treated with IL‐1β, with or without E2, are shown. (d) Western blotting was conducted to assess the expression of p16 and p21 in chondrocytes after treatment with H_2_O_2_, with or without E2 (*n* = 4). (e, f) The relative levels of p16 (e) and p21 (f) in chondrocytes after treatment with H_2_O_2_, with or without E2. (g) Western blotting was performed to assess the expression of p16 and p21 in chondrocytes exposed to 0 or 5 Gy ion radiation with or without E2 (*n* = 4). (h, i) The relative levels of p16 (h) and p21 (i) in chondrocytes exposed to 0 or 5 Gy of ion radiation, with or without E2, are shown. (j) Western blotting was conducted to assess the expression of p16 and p21 in chondrocytes treated with etoposide (ETO), with or without E2 (*n* = 4). (k, l) The relative levels of p16 (k) and p21 (l) in chondrocytes treated with etoposide (ETO), with or without E2, are provided. One‐way analysis of variance (ANOVA) was applied, followed by Bonferroni's post hoc test for multiple comparisons. A *p*‐value of less than 0.05 was considered statistically significant. All the data are presented as the mean ± SD. *p < 0.05, **p < 0.01, ***p < 0.001.

Cellular senescence is a complicated biological process that can be induced by various factors. After finding that E2 could counteract chondrocyte senescence induced by IL‐6, we attempted to determine whether E2 exerts an antisenescence effect in other types of cellular senescence. In the following experiments, we used normal chondrocytes and different senescence inducers. H_2_O_2_ was used to model oxidative stress‐induced senescence, IL‐1β was used to model inflammation‐induced senescence, and etoposide was used to model DNA damage‐induced senescence. Ionising radiation (5 Gy) was also used to model DNA damage‐induced senescence. Under IL‐1β treatment conditions, the expression of p16 and p21 was increased compared with that in the control group. However, when E2 was added, the expression of p16 and p21 returned to normal levels, compared with the IL‐1β treatment group (Figure 6a–c). For the H_2_O_2_‐induced senescence (Figure 6d–f), the results were similar. However, neither IR‐induced senescence (Figure 6g–i) nor etoposide‐induced senescence (Figure 6j–l) could be counteracted by E2 treatment.

### 3.8 Intra‐Articular Injection of E2 Delayed Rat OA Progression

**FIGURE 7:**
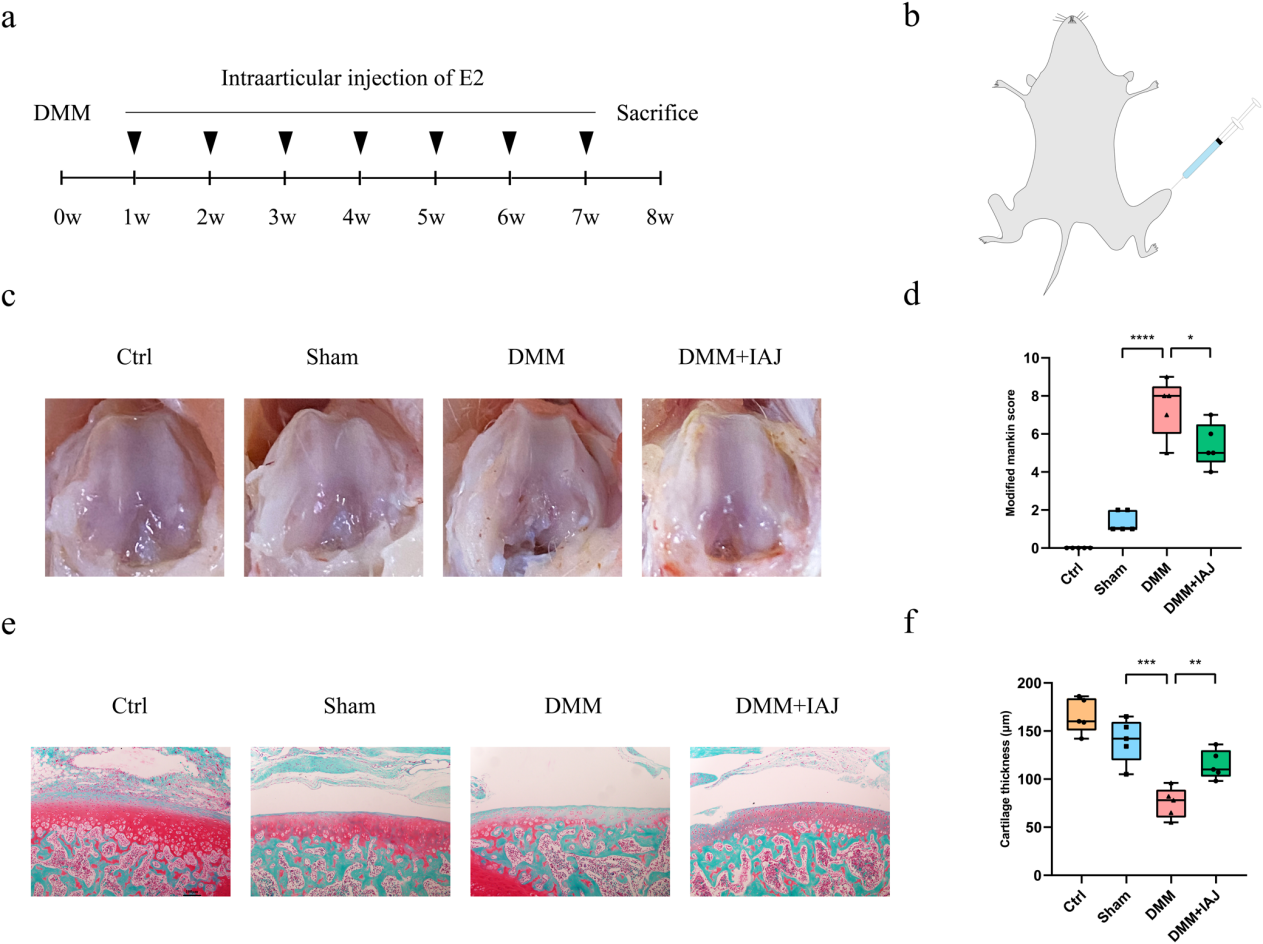
Intra‐articular injection of E2 ameliorated OA progression in rats. (a) Timeline of the rat experiments. (b) Illustration of intra‐articular injection in the rat. (c) Gross observation of rat knees after sacrifice from the control group (Ctrl), sham surgery group (Sham), DMM surgery group (DMM), and DMM surgery with weekly postoperative intra‐articular injection group (DMM + IAJ) (*n* = 5 per group). (d) Modified Mankin scores of the knees are shown. (e) Representative Safranin O staining of knees under a high‐power microscope (100×) is presented. (f) Cartilage thickness of the knees is measured. DMM: Destabilise the medial meniscus; IAJ: Intra‐articular injection. One‐way analysis of variance (ANOVA) was applied, followed by Bonferroni's post hoc test for multiple comparisons. A *p*‐value of less than 0.05 was considered statistically significant. All the data are presented as the mean ± SD. *p < 0.05, **p < 0.01, ***p < 0.001, ****p < 0.0001.

We performed in vivo experiments to evaluate the efficacy of E2 in ameliorating OA. Surgery to destabilise the medial meniscus (DMM) was performed on rats at 12 weeks of age to establish the OA model, and the knees were harvested at 20 weeks of age (Figure 7a). E2 was injected into the knee joint cavity of the rats once per week after surgery to ensure a long‐term and sufficient dosing of E2 therapy. In contrast, normal saline was administered to the sham and control groups instead (Figure 7b). Based on the gross knee specimens, there was severe damage to the articular surfaces in the DMM group. The cartilage surface was rough, and cartilage collapse was visible in most wear and tear regions. Cartilage in the DMM + IAJ group had a better appearance compared with the DMM group, and that in the sham group and control group was almost intact (Figure 7c). The DMM + IAJ group exhibited better modified Mankin's score (Figure 7d) and cartilage thickness (Figure 7e,f) than the DMM group. No severe adverse outcomes were observed after 7 rounds of E2 administrations. These findings confirmed the protective effects of E2 in the DMM‐induced rat OA model.

## 4 Discussion

In recent years, numerous studies have reported the crucial role of chondrocyte senescence in OA development, and researchers have begun to consider chondrocyte senescence as a therapeutic target [10]. Preventing chondrocyte senescence to ameliorate OA progression is promising. The pathogenesis of OA is complex and diverse, with multiple factors such as aging, oestrogen withdrawal, mechanical stress overload, and metabolic disorders causing cartilage degeneration and OA development [49]. The existing treatments for OA mainly focus on relieving pain, and there are few drugs to control OA progression. Therefore, identifying new therapeutic targets for OA and elucidating the role of senescence in its pathogenesis are essential.

In our study, we confirmed the negative influence of FOXO4 on chondrocyte senescence and demonstrated the protective effect of E2 against cellular senescence. The downstream protein FOXO4 responded to AKT signalling and was degraded under E2 treatment conditions, thereby inhibiting the subsequent biological processes. We established the ERα‐AKT‐FOXO4 axis in chondrocytes and demonstrated that E2, for the first time, could be used as an anti‐senescence agent in the treatment of OA.

We found increased expression of FOXO4 in osteoarthritic cartilage and in vitro expanded chondrocytes that underwent IL‐6 treatment. Our results are supported by another research, which revealed higher levels of FOXO4 mRNA and protein in natural degenerative (16‐week‐old rats) or induced degenerative (IL‐1β‐treated chondrocytes from 4‐week‐old rats) rat cartilage [50]. Moreover, we found that FOXO4 played an important role in promoting chondrocyte senescence during OA progression, which is consistent with previous studies [24, 25]. Reduced FOXO4 phosphorylation is associated with its stabilisation. In contrast, increased phosphorylation triggers its nuclear export, leading to subsequent degradation. Serrano et al. [51] reported a harmful effect of decreased levels of phosphorylated FOXO4 and revealed that it promoted impaired proteasomal function in OA chondrocytes, dysregulation of chondrocyte homeostasis, and decreased levels of SOX9 mRNA and protein. We found an increased level of phosphorylated FOXO4 after E2 treatment, which may explain the protective effect of E2 against chondrocyte senescence and OA progression.

We demonstrated that E2 activates AKT, thereby inducing FOXO4 degradation. Multiple studies underscore the anti‐senescence role of the PI3K‐Akt pathway across various biological contexts. For instance, it has been shown to inhibit senescence and promote self‐renewal in human skin‐derived precursors by modulating FoxO3 and GSK‐3β [52]. Similarly, AKT activation can overcome RAS‐induced senescence to facilitate tumorigenesis [53]. Beyond its anti‐senescence function, restoring AKT activity has been demonstrated to alleviate age‐related muscle loss [54]. Moreover, a more recent mechanism reveals that AKT phosphorylates GIRDIN at Ser1417, thereby enhancing platelet phagocytosis and delaying cellular aging [55]. However, given the extensive downstream signalling of AKT, FOXO4 is unlikely to be the only molecule responsible for the anti‐senescent effects of E2. Other key AKT downstream molecules, such as mTOR—which has been implicated in alleviating senescence—are likely involved. This is supported by findings that Panax notoginseng saponins counteract senescence in osteoarthritic chondrocytes via the PI3K‐AKT–mTOR pathway [56].

There are three common receptors of E2: ERα and ERβ, which form either homo‐ or heterodimers when bound to E2. These dimers then bind to the oestrogen responsive element to regulate related gene transcription [57]. Additionally, GPER is a G protein‐coupled receptor located in the membrane that induces rapid responses when activated [58]. Our previous studies revealed that E2 inhibited chondrocyte apoptosis via GPER activation [28]. Initially, we hypothesised that the antisenescence effect of E2 may also occur via GPER, as GPER is usually associated with rapid responses to E2. However, when GPER was blocked with G‐15, no obvious changes in the levels of p16 and p21 were observed. In contrast, blocking or knocking down ERα counteracted the antisenescence effect of E2. Therefore, we concluded that ERα, not ERβ or GPER, mediated the protective effect of E2 against cellular senescence in chondrocytes. It is also plausible that ERα translocates to the membrane and mediates a rapid response [57], which could explain our results.

We established a surgery‐induced rat model to test the efficacy of E2 in preventing the progression of OA. We considered both systemic and local administration. The half‐life of E2 in systemic circulation is relatively short after systemic administration. This rapid clearance necessitates the use of higher doses to maintain effective E2 concentrations in the joints for ideal cartilage protection. However, high‐dose systemic E2 can lead to significant side effects and compromise the health of the animals. Therefore, we adopted an intra‐articular injection strategy to overcome the rapid systemic clearance and minimise potential side effects by establishing a local “reservoir” of E2 within the joint cavity [59]. Although the exact half‐life of E2 in rat joints remains unclear, it is widely accepted that locally administered drugs exhibit considerably longer retention times compared to those in the bloodstream. A weekly dose of 5 μg/kg was selected to achieve an initially high local concentration and a sustained therapeutic level of E2 in joint tissue. Our results suggested that E2 ameliorated surgery‐induced OA.

Taken together, these results revealed a protective effect of E2 against several types of chondrocyte senescence. This effect was mediated by ERα, and the downstream activation of AKT and subsequent degradation of FOXO4 played vital roles in this process. Furthermore, for the first time, we demonstrated the efficacy of E2 in the treatment of OA using a rat model. Our study reveals that E2 protects cartilage by preventing chondrocyte senescence, thereby offering new directions for the developing DMARDs against OA. Additionally, it provides a preliminary research basis for employing oestrogen replacement therapy to prevent and treat OA in postmenopausal women.

## Author Contributions

**Yikai Liu:** writing – original draft, investigation, project administration, data curation. **Jiangshan Ai:** writing – original draft, project administration, investigation. **Zian Zhang:** writing – original draft, data curation, formal analysis. **Xinzhe Lu:** writing – original draft, formal analysis. **Chaoqun Yu:** writing – original draft, formal analysis. **Yejun Zha:** writing – review and editing, funding, supervision, validation. **Haining Zhang:** writing – review and editing, funding, supervision, validation.

## Funding

This work was supported by the Horizontal Project: A Prospective Randomised Controlled Study on the Effect of Jintiange on Quadriceps Muscle Atrophy and Muscle Strength Improvement in Patients After Total Knee Arthroplasty; Henan Academy of Medical Sciences “Three 100s” Program (Clinical Research Physician Track No. HNCRD202439); National Natural Science Foundation of China (No. 81672197); Beijing Municipal Public Welfare Development and Reform Pilot Project for Medical Research Institutes (JYY2023‐11); National Key R&D Program of China (2024YFC3044700).

## Ethics Statement

This study was approved by the medical ethics committee of the Affiliated Hospital of Qingdao University (Approval No. QYFY WZLL 27764). Written informed consent was obtained from all participants.

## Consent

The authors have nothing to report.

## Conflicts of Interest

The authors declare no conflicts of interest.

## Supporting information


**Figure S1:** E2 could alleviate IL‐6‐induced chondrocyte senescence. (a) Immunofluorescence staining of γ‐H2AX was performed after treatment with IL‐6 and/or E2 (*n* = 3). (b) The relative expression of γ‐H2AX is shown. (c) The concentration of MMP‐13 in the supernatant of the culture medium was measured by ELISA. One‐way analysis of variance (ANOVA) was applied, followed by Bonferroni's post hoc test for multiple comparisons. A *p*‐value of less than 0.05 was considered statistically significant. All data are presented as the mean ± SD. * *p* < 0.05, **p < 0.01.

**Figure S2:** E2 promoted the nuclear export of FOXO4. (a) Western blotting of nuclear and cytoplasmic fractions was performed to assess FOXO4 subcellular localisation. (b, c) The relative levels of FOXO4 in the nucleus and cytoplasm are shown. One‐way analysis of variance (ANOVA) was applied, followed by Bonferroni's post hoc test for multiple comparisons. A *p*‐value of less than 0.05 was considered statistically significant. All data are presented as the mean ± SD. * *p* < 0.05, **p < 0.01.

**Figure S3:** The dynamics of FOXO4 degradation were assessed by cycloheximide (CHX) chase assay. (a) Western blotting analysis of FOXO4 was performed at 0, 2, and 4 h after CHX treatment in the presence of E2 and/or MG‐132. MG‐132 and E2 were added 4 and 2 h before CHX, respectively, to ensure complete proteasome inhibition and initiation of E2 signalling prior to degradation tracking. (b) The relative levels of FOXO4 are shown. One‐way analysis of variance (ANOVA) was applied, followed by Bonferroni's post hoc test for multiple comparisons. A *p*‐value of less than 0.05 was considered statistically significant. All data are presented as the mean ± SD. * *p* < 0.05.

## Data Availability

The data that support the findings of this study are available from the corresponding author upon reasonable request.
