# Supplementary figures and images for: 17‐β‐Estradiol Protects Chondrocytes From Senescence and Ameliorates Osteoarthritis Progression via ERα‐AKT‐FOXO4 Pathway
Source: J Cell Mol Med. 2026 Feb 12;30(3):e71018. doi: 10.1111/jcmm.71018 (PMC12895378; doi:10.1111/jcmm.71018)

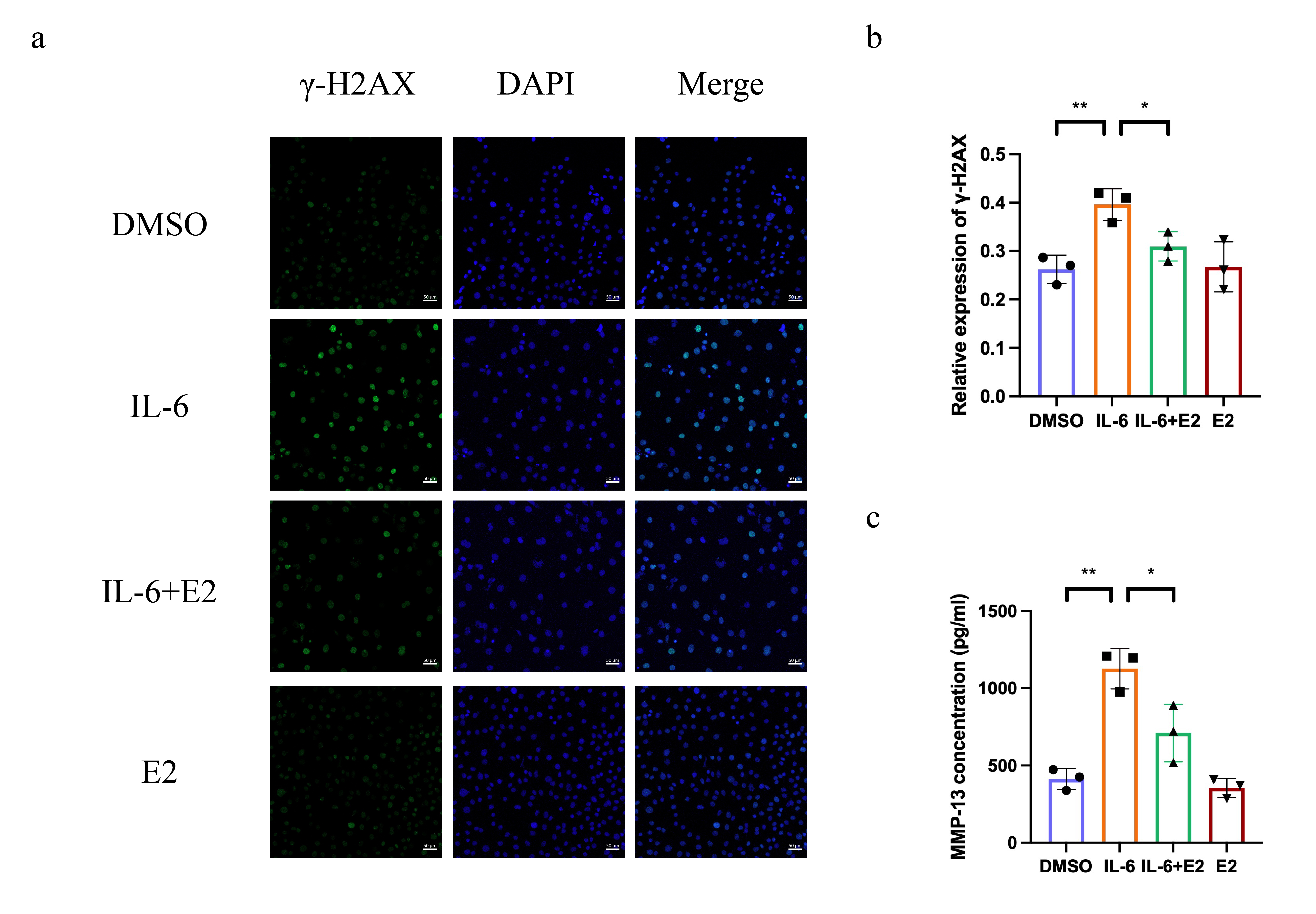

Supplement: Supplementary file 1 — Figure S1: E2 could alleviate IL‐6‐induced chondrocyte senescence. (a) Immunofluorescence staining of γ‐H2AX was performed after treatment with IL‐6 and/or E2 (n = 3). (b) The relative expression of γ‐H2AX is shown. (c) The concentration of MMP‐13 in the supernatant of the culture medium was measured by ELISA. One‐way analysis of variance (ANOVA) was applied, followed by Bonferroni's post hoc test for multiple comparisons. A p‐value of less than 0.05 was considered statistically significant. All data are presented as the mean ± SD. * p < 0.05, **p < 0.01. [file JCMM-30-e71018-s002.jpg]

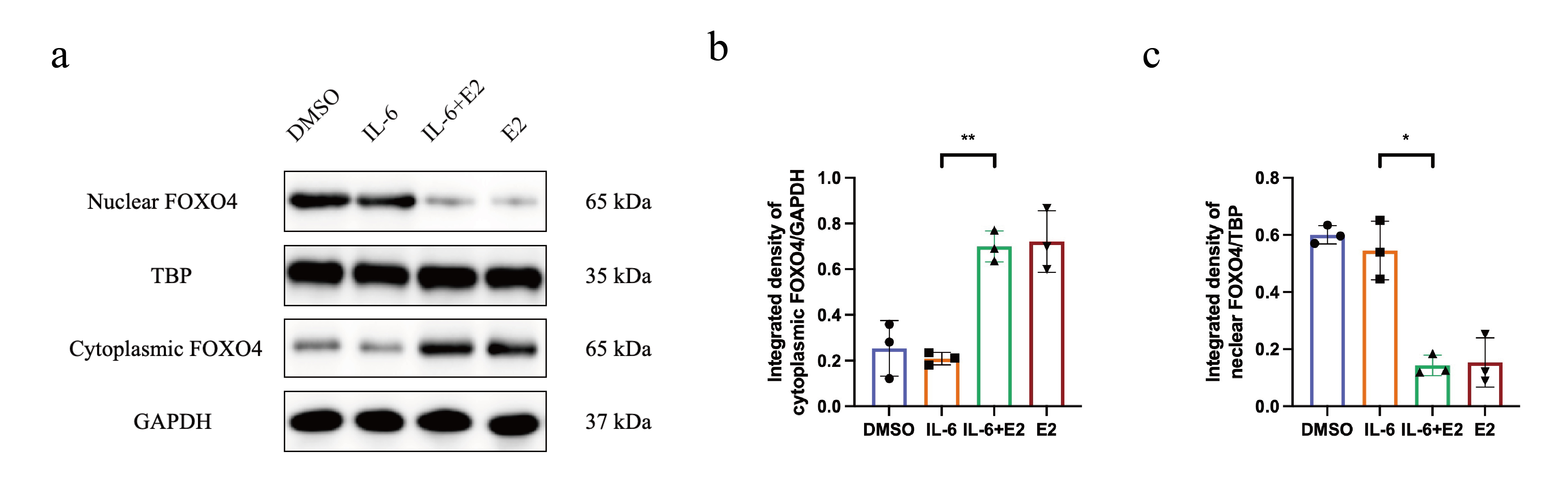

Supplement: Supplementary file 2 — Figure S2: E2 promoted the nuclear export of FOXO4. (a) Western blotting of nuclear and cytoplasmic fractions was performed to assess FOXO4 subcellular localisation. (b, c) The relative levels of FOXO4 in the nucleus and cytoplasm are shown. One‐way analysis of variance (ANOVA) was applied, followed by Bonferroni's post hoc test for multiple comparisons. A p‐value of less than 0.05 was considered statistically significant. All data are presented as the mean ± SD. * p < 0.05, **p < 0.01. [file JCMM-30-e71018-s003.jpg]

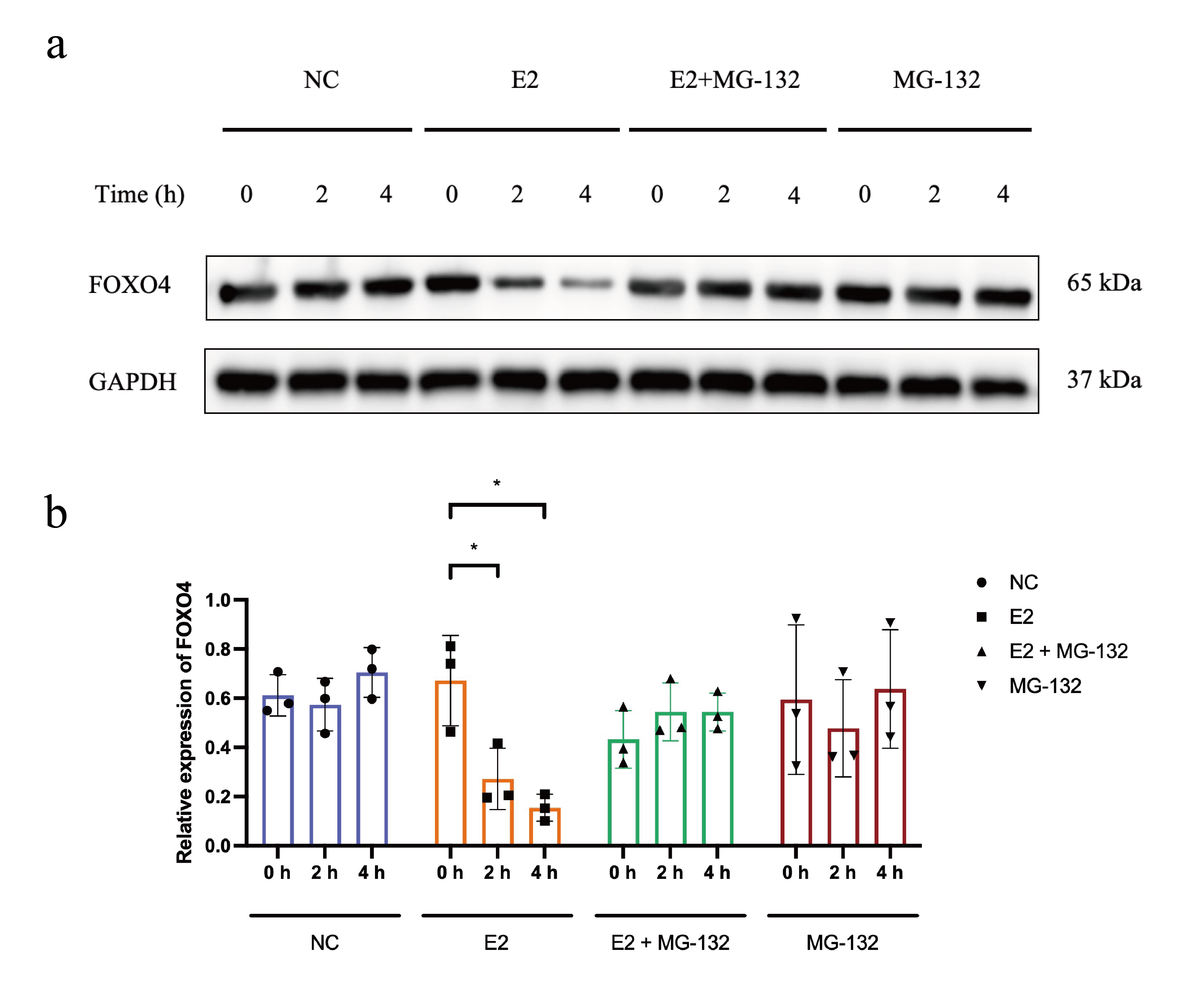

Supplement: Supplementary file 3 — Figure S3: The dynamics of FOXO4 degradation were assessed by cycloheximide (CHX) chase assay. (a) Western blotting analysis of FOXO4 was performed at 0, 2, and 4 h after CHX treatment in the presence of E2 and/or MG‐132. MG‐132 and E2 were added 4 and 2 h before CHX, respectively, to ensure complete proteasome inhibition and initiation of E2 signalling prior to degradation tracking. (b) The relative levels of FOXO4 are shown. One‐way analysis of variance (ANOVA) was applied, followed by Bonferroni's post hoc test for multiple comparisons. A p‐value of less than 0.05 was considered statistically significant. All data are presented as the mean ± SD. * p < 0.05. [file JCMM-30-e71018-s001.jpg]
